# Win-Win: Anthropogenic circularity for metal criticality and carbon neutrality

**DOI:** 10.1007/s11783-023-1623-2

**Published:** 2022-09-05

**Authors:** Xianlai Zeng

**Affiliations:** State Key Joint Laboratory of Environment Simulation and Pollution Control, School of Environment, Tsinghua University, Beijing, 100084 China

**Keywords:** Anthropogenic circularity, Material flow analysis, Criticality, Carbon neutrality, Solid waste, Circular economy

## Abstract

Resource depletion and environmental degradation have fueled a burgeoning discipline of anthropogenic circularity since the 2010s. It generally consists of waste reuse, remanufacturing, recycling, and recovery. Circular economy and “zero-waste” cities are sweeping the globe in their current practices to address the world’s grand concerns linked to resources, the environment, and industry. Meanwhile, metal criticality and carbon neutrality, which have become increasingly popular in recent years, denote the material’s feature and state, respectively. The goal of this article is to determine how circularity, criticality, and neutrality are related. Upscale anthropogenic circularity has the potential to expand the metal supply and, as a result, reduce metal criticality. China barely accomplished 15 % of its potential emission reduction by recycling iron, copper, and aluminum. Anthropogenic circularity has a lot of room to achieve a win-win objective, which is to reduce metal criticality while also achieving carbon neutrality in a near closed-loop cycle. Major barriers or challenges for conducting anthropogenic circularity are deriving from the inadequacy of life-cycle insight governance and the emergence of anthropogenic circularity discipline. Material flow analysis and life cycle assessment are the central methodologies to identify the hidden problems. Mineral processing and smelting, as well as end-of-life management, are indicated as critical priority areas for enhancing anthropogenic circularity.

**Electronic Supplementary Material:**

Supplementary material is available in the online version of this article at 10.1007/s11783-023-1623-2 and is accessible for authorized users.

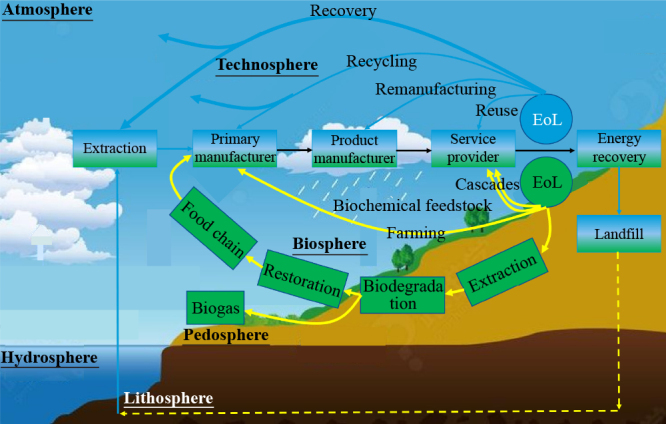

## 1 Introduction

Our humans began to drastically affect the earth after the Industrial Revolution, particularly after 1945’s “Great

✉ Corresponding author
E-mail: xlzeng@tsinghua.edu.cn
Special Issue—Young Talent

Acceleration," and this age is known as the Anthropocene (Steffen et al., 2015). Improved earth system governance necessitates changes in citizen behavior, increased civil society engagement, and a shift in the private sector toward a green economy (Biermann et al., 2012). Over the last century, the carbon, nitrogen, and phosphorus cycles have been significantly altered (Gruber and Galloway, 2008; Liu et al., 2016a; Waters et al., 2016).

Unsustainable exploitation and usage of natural resources have been crucial to our economic progress. Over the last 50 years, global material resource extraction has quadrupled, and it is predicted to at least double with the rise of developing nations (IRP, 2019). We exploit and utilize our planet's resources much quicker than they can be replenished today. Changing to a low-carbon economy and energy system, on the other hand, may raise demand for some important minerals and strategic resources. Copper use, for example, will peak between 2030 and 2040 (Kerr, 2014). Overall, many geological metal minerals are in poor supply or perhaps depleted.

Environmental pollution is also an important issue linked to resource consumption and scarcity. The release of materials and substances into the environment causes pollution. Solid waste has been exploding at a breakneck pace. In 2010, the global generation data for municipal solid waste and industrial & construction solid waste was 2 billion tons and 7–10 billion tons (1 ton = 1000 kg), respectively (UNEP and ISWA, 2015). Only China produced 10–12 billion tons of solid waste in 2018, with 60–70 billion tons dumped without being disposed of. This century will see a peak in global waste output (Hoornweg et al., 2013). Global demand for environmental measurement is increasing, and newly amended legislation are allowing costs to be internalized (Hodges, 1995; Liang et al., 2021).

Here, I choose the metal to examine the performance of resource shortage and waste explosion. Metal-production sector was responsible for 7.9 % of global carbon emissions (Lamb et al., 2021). Metal recycling and technical advancements will help to maintain supply, but mining must continue and expand in the near future to ensure that such minerals are available to industry (Ali et al., 2017). Primary and secondary metals must be viewed as parts of a same system that must be fully comprehended (Bloodworth, 2014). Driven by the concept of recycling, anthropogenic circularity (see the definition in Appendix A) science is an emerging area from the interdisciplinary development of earth science, technology science, and management science (Zeng and Li, 2021). Only a few works mentioned the circularity and carbon neutrality (Al-Obaidy et al., 2021; Mangold and von Vacano, 2022). No found publications addressed the anthropogenic circularity performance on metal criticality and carbon emission. This work, therefore, will analyze the scientific link between metal consumption and carbon emission in the context of carbon neutrality, and measure the win-win condition of anthropogenic circularity for metal criticality and carbon neutrality.

## 2 Framework and theory of anthropogenic circularity

### 2.1 Framework of anthropogenic circularity

While subsurface minerals are extracted and mined for fine metals or alloys, products are developed and manufactured (e.g., durable goods). Few compounds escape into the hydrosphere, atmosphere, and pedosphere along the entire material movement, potentially contaminating water, air, and soil. The bulk of material eventually becomes solid waste: one portion is produced as tailings, slag, home scrap, and new scrap as a result of extraction, production, or manufacture, while the other part is produced as product waste or old scrap as a result of end-of-life (EoL) consumption. Some well-used products can be reused, some old product can be remanufactured after disassembly, most broken products will be dismantled for recycling, and litter will be recovered for a closed-loop or open-loop as other product supply chains (Fig. 1).

### 2.2 Theory of anthropogenic circularity

Anthropogenic circularity, driven by human activities and natural geodynamics, is reshaping the biogeochemical cycle in the anthroposphere (or technosphere) (Rauch and Graedel, 2007). Natural biogeochemistry and social-economy metabolism, such as trade and logistics, make

**Fig. 1** Framework of anthropogenic circularity along the material flow (Note: revised from Zeng and Li, 2021).

up material flow in the macroworld (Baccini and Brunner, 2012; Krausmann et al., 2018). From the global insight, the spatial landscape of typical metals has changed dramatically since 1960. As anthropogenic stock, geological metals minerals, for example, have been dramatically removed from subsurface to aboveground (i.e., in-use stock and waste). Currently, the twelve most common metals, such as Ag, Au, Bi, Cd, Cu, Fe, Hg, In, Pd, Sb, Sn, and Zn, have a visible stock on the surface, indicating that the bulk of these metals have been extracted from the geological reserve (Fig. 2). While anthropogenic stock of these metals in 2020 accounted for over 70 % of total geological and anthropogenic resource, some geological metals like In (88 %), Cd (87 %), Hg (84 %), Sb (82 %), Sn (76 %), Au (74 %), and Pb (73 %), are rapidly depleting. But China demonstrated a more severe situation than global circumstance, in particular on Co, Pb, Ni, and Al (Eheliyagoda et al., 2022; Zhou et al., 2022). Furthermore, trade plays an important function in balancing the regional landscape. China, for example, is the global manufacturing hub because it imports resources from Australia and exports the finished product to the United States. Individual mining, manufacturing, and recycling companies might pursue resource utilization and waste minimization in the microworld.

Three laws of anthropogenic circularity science have been summarized by Ref. (Zeng and Li, 2021) and derived from Ref. (Jin, 2014). According to the first law of anthropogenic circularity, the relative contents (or abundance) of different elements in the cosmos are constant. It discusses the significance and scope of human-caused circularity. Natural resources on Earth are finite, therefore material supply from circularity is critical to alleviate the scarcity of virgin minerals. The principle of material cycle is the second law of anthropogenic circularity. In an ecological world, this means that all materials and products generated from them circulate along cyclical channels. During the entire cycle, material can be altered in form but never vanishes. It shows that recycling is possible. Finally, the notion of zero waste is the third law of anthropogenic circularity. The biogeological material cycle is always a near-zero-emission process. In an ideal world, anthropogenic circularity

**Fig. 2** The global evolution of spatial landscape of typical metals in 1970–2020. Note: Data of geological reserve source from USGS. Anthropogenic stock, consisting of in-use, waste, and even landfill, is the sum of previous production (1 Tt = $10^3$ Gt = $10^6$ kt = $10^9$ t).

along with some chemical reactions should be tailored to achieve zero waste emission.

### 2.3 Metric of anthropogenic circularity

To measure anthropogenic circularity, humans just started to establish the metric or indicator system. Until now, there is still no standardized method to determine the circularity (Linder et al., 2017). Anthropogenic circularity could be measured at different spatial scales, ranging from macro- (national or regional) and meso- (industrial park), to micro-levels (corporation) with various metrics or indicators. For instance, recycling rate and resource efficiency are commonly employed to measure the circular economy (Bîrgovan et al., 2022). The recycling rate and detoxification rate are used to examine waste management. Emergy analysis is an emerging metric used for an industrial park (Liu et al., 2016b) and industry system (Song et al., 2013). Recyclability is adopted to measure the recycling difficulty of solid waste and helps in product eco-design (Anctil and Fthenakis, 2013; Kanwal et al., 2021; Teseletso and Adachi, 2022). A circular economy indicator system was proposed to measure the progress of one country or city (Bîrgovan et al., 2022; Geng et al., 2019). On this basis, the method of Multiple Correspondence Analysis was proposed to examine 63 circularity metrics and 24 features relevant to the circular economy, such as recycling rate, resource productivity, and disposal rate (Parchomenko et al., 2019). Ruiz-Pastor et al. (2022) designed a metric, CN_Con, to measure the circularity. It will help to compare quantitatively the circularity and novelty potential of different design alternatives (Ruiz-Pastor et al., 2022).

## 3 Anthropogenic circularities against metal criticality

### 3.1 Metal criticality and its measurement

Massive metals have been seized by the rapid advancement of high technology. According to O’Connor et al. (2016), printed circuit boards comprised only eleven elements in the 1980s, fifteen in the 1990s, and sixty in the 2000s (O’Connor et al., 2016). Rare metals are increasingly being used in high-tech items in contemporary culture. The emergence of criticality was prompted by shortage. Since 2010, the term “criticality” has been formalized to define the quality, state, or degree of being of the utmost importance, and it has recently sparked interest in strategic metals (Graedel and Nuss, 2014). Prof. T. E. Graedel at Yale University proposed and created the measurement model of metal criticality. Criticality indicates the significance of metal from two dimensions of the supply risk and the vulnerability (Erdmann and Graedel, 2011) to three dimensions of the supply risk, the environmental implications, and the vulnerability to supply restriction (Graedel et al., 2012). The economy, geology, commerce, regulation, policy, and even politics are all variables in the three dimensions. It’s calculated using three dimensions and dynamic weight.

Numerous resources have been investigated, including copper family (Nassar et al., 2012), iron & alloy (Nuss et al., 2014b), metalloids (Graedel et al., 2015), nuclear energy metals (Harper et al., 2015a), zinc, tin, & lead (Harper et al., 2015b), rare earth element (Nassar et al., 2015), water (Sonderegger et al., 2015), and specialty metals (Panousi et al., 2016). Precious metals have the most serious environmental consequences, even when compared to some ordinary metals. Because of the dynamic nature and variance, criticality differs from nation to country (Glöser-Chahoud et al., 2016; Ciacci et al., 2017). Until March 2022, over 50 articles on criticality were published, covering over 60 base metal, rare metal, and rare earth elements. Copper, indium, niobium, and a few precious metals are among the most concentrated metals (Fig. 3). It suggests that in many countries, these metals are a top priority among all critical metals.

### 3.2 Relationship between anthropogenic circularity and metal criticality

In recent years, many studies on criticality have been translated into some applications to guide the automobile industry (Knobloch et al., 2018), the thin-film photovoltaic technologies (Zhou et al., 2020), and transport network (Colon et al., 2021). China is also highlighting the critical metals issue along the whole supply chain (Wang et al., 2019). Tsinghua University established the measurement method of carrying capacity for critical metals based upon the closed-loop supply chain and completed over ten critical metals for China. In 2019, the National Natural Science Foundation of China initiated a major research plan related to critical metals. It is devoted to answering three key scientific questions: the multi-layer interaction of the earth and the enrichment process of critical metals, the metallogenic mechanism of critical metals, and the occurrence state and enhanced separation mechanism (Hou et al., 2020). The three-dimensional framework was adopted to measure the copper and aluminum in the world (Eheliyagoda et al., 2020). The values of platinum-group metals in the three dimensions were reported to rank in the first two places, indicating a higher supply shortage risk as well as a vital and fundamental role for the Chinese economy and facilities construction (Yan et al., 2021).

Anthropogenic circularity can offer certain resources for industry and minimize the demand for primary (or virgin) metal through reuse, remanufacturing, recycling,

**Fig. 3** Periodic table of metal criticality and global warming potentials (GWPs). Note: color indicates publications amount on criticality, and data indicates GWPs of mining in kg $CO_2$-eq/kg. Publications amount, Table S1 of Supplementary Material, was searched from Web of Science and data source of GWPs from Ref. Nuss and Eckelman (2014a).

and recovery. As a result of design for circularity, increasing product recyclability and longevity is also a key signal of criticality measurement. It thereby may be able to reduce metal demand supply risk and ease metal criticality (Babbitt et al., 2021). In the case of rare earth elements, for example, the production of scrap was small in the short term compared to the expanding demand. However, one efficient method of combating criticality has been established as anthropogenic circularity through recycling (Rademaker et al., 2013). A large amount of energy metals such as lithium, cobalt, indium, nickel, and gallium have been consumed in the new energy business (Olivetti and Cullen, 2018). The associated circular business models can be employed to reduce material criticality (Velenturf et al., 2021). Hence, elevating anthropogenic circularity can bring down material criticality (Tercero Espinoza et al., 2020).

## 4 Anthropogenic circularities for carbon neutrality

### 4.1 Carbon neutrality and its measurement

From the dictionary, neutrality in chemical science is the condition of being chemically or electrically neutral. Carbon neutrality is defined as an environmental carbon balance sheet with zero net $CO_2$ equivalents ($CO_{2eq}$) emissions. Carbon neutrality refers to net-zero $CO_2$ emissions attained by balancing the emission of $CO_2$ with its removal so as to stop its increase in the atmosphere that causes global warming. For the aluminum industry, carbon neutrality is defined as a state where the total "in-use" $CO_{2eq}$ saved from all products in current use, including incremental process efficiency improvements, collection, and urban mining activities, equals the $CO_{2eq}$ extended to produce the global output of aluminum (Das, 2012; Raabe et al., 2019).

In the 21st century, carbon neutrality is becoming the new industry paradigm around the world. Carbon neutrality may be regarded as the fifth industrial revolution. As of February 2021, 124 countries had pledged to achieve carbon neutrality by 2050 or 2060. Confronting the urgent need to deal with climate change, the "Copenhagen Accord" and the "Paris Agreement" propelled governments to establish domestic targets to reduce carbon emissions. Its measurement is an emerging and hot issue in the world. A spurt of publication is addressing this issue. At least in China, carbon accounting is entering the industrial sectors and indicates a coming massive movement of carbon emission reduction.

### 4.2 Relationship between anthropogenic circularity and carbon neutrality

Because recycling usually uses less energy than creating new materials, increasing circularity reduces pollution while also lowering energy demand (Iranpour et al., 1999; Lu et al., 2022). Metal primary mining's cumulative energy requirement has a considerable impact on global warming potential (Fig. 4(a)). Higher energy demand apparently indicates higher global warming potential since all forty-three metals have a linear relationship. As a consequence, increasing circularity has the potential to reduce $CO_2$ emissions. For example, the primary and recycling $CO_2$ emissions for a one-ton copper mine are roughly 1.71 and 0.88 t, respectively (Fig. 4(b)). Precious metals such as gold and palladium have a massive discrepancy in absolute value. But the aluminum and iron

**Fig. 4** (a) Global warming potential of primary mining; (b) $CO_2$ equivalent emission of primary and recycling process for typical metals. Note: Data source from refs. (Norgate and Haque, 2010; USEPA, 2012; Ashby and Johnson, 2014; Liu, 2014; Nuss and Eckelman, 2014a; Wang et al., 2022); Ratio (%) = Recycling / Primary process × 100. For instance, the $CO_2$ emission of Cu was 1.71 and 0.88 kg per kg metal yield for primary process and recycled process, respectively. The ratio of Cu will be 51 %.

have a lower ratio so they have a comparatively high potential for $CO_2$ emission reduction.

Similar to metal, plastic circularity can provide environmental benefits through a circular economy. Klotz and his colleagues built the material flow system of 11 plastic types in 54 products in Switzerland in 2017 and found that 21 % to 100 % of the secondary material gained can substitute 11 % to 17 % of the total material demand (Klotz et al., 2022). Pulling valuable metals from anthropogenic mineral makes financial sense over virgin mining of geological mineral (Zeng et al., 2022), which could be subject to low energy input and less carbon emission in a circular economy (Azadi et al., 2020; Eheliyagoda et al., 2022). Critical metals, on the other hand, are necessary to support carbon-neutral decarbonization technologies such as solar and wind energy (Velenturf et al., 2021).

## 5 Practice and opportunities of anthropogenic circularity

### 5.1 Practice of anthropogenic circularity

At the practical level, anthropogenic circularity has been enabled. Waste recycling, clean production, circular economy, urban mining, and a zero-waste program are all common activities around the world (Zeng and Li, 2021). Material flow analysis can indicate the progress of anthropogenic circularity (Brunner and Rechberger, 2016). Copper, for instance, is one of the most important metals to function in our high-tech industry and society. Local copper is fed after the excavation from the lithosphere and importation from other places. The fine copper is used for fabrication and manufacturing (F&M). After the consumption, the product will reach the EoL and become the product waste (or old scrap). Some waste generated in the production or F&M stages covers tailings, slag, and defective goods (or new scrap), of which the new scrap is much easier than tailings and slag in metal recycling. At the EoL stage, the old scrap like e-waste can be remanufactured and recycled, and a part as loss flows to a landfill (Charpentier Poncelet et al., 2022).

Copper is mostly consumed in Europe, China, Japan, and the United States. To show copper flow practice, two time periods in the mid-1990s and the 2010s were chosen (Fig. 5). Total geological mineral extraction for production was roughly 6.72 Mt (1 Mt = $10^6$ t) from the lithosphere and 5.25 Mt from importation. Only 4.35 Mt of anthropogenic mineral was used in production, with 12 % coming from old scrap and 88 % from new scrap. Only 35 % of demand could be met by recycling anthropogenic minerals through a circular economy. About 15.03 Mt of old scrap was buried as loss: 13 %, 25 %, and 15 % of it went to remanufacturing, recycling, and landfilling, respectively, while the rest (47 %) was not collected or handled formally. From the 1990s to the 2010s, no substantial differences were observed in Europe, Japan, or the United States. In each flow, China displayed a remarkable growth. We are still a long way from a closed-loop circularity system on a global scale (Reck and Graedel, 2012).

### 5.2 Influence of anthropogenic circularity

An increase in anthropogenic circularity can help to assure metal supply security while also lowering criticality. Here using the existing method of metal criticality, I quantify the influence of anthropogenic circularity upon criticality in China. Copper and aluminum were enabled by Eq. (1). If no recycling, the criticality of copper and aluminum is 46 % and 39 %,

**Fig. 5** Substance flow diagram of copper in some countries. Note: stock data is not indicated here. Data source of Europe 1994 from Refs. (Rechberger and Graedel, 2002; Spatari et al., 2002); Europe 2014 from Refs. (Ciacci et al., 2017; Soulier et al., 2018); China 1994 from Ref. (Graedel et al., 2004); China 2010 from Ref. (Zhang et al., 2014); Japan 1994 from Ref. (Kapur et al., 2003); Japan 2005 from Ref. (Daigo et al., 2009); and U.S. 1995 and 2012 from Refs. (Spatari et al., 2005; Wang et al., 2015; Chen et al., 2016). The sum of flows is also given in this figure. For instance, 6.72 from lithosphere to production is the total flow of Europe, China, Japan, and the U.S. for the two years.

respectively. With the Chinese real recycling situation, the criticality of copper and aluminum is 47 % and 41 %, respectively. In the case of full recycling, their criticalities reach 51 % and 44 %. Thus, China had 4 % and 3 % of the utmost increase potential of anthropogenic circularity for the criticality of copper and aluminum, respectively (Fig. 6(a)).

$$C=\sqrt{\frac{SR^2+ER^2+SRR^2}{3}}=\begin{cases}\sqrt{\dfrac{46^2+36^2+[(373+RR)/7]^2}{3}}=\sqrt{\dfrac{3412+[(373+RR)/7]^2}{3}} & \text{Cu}\\[2ex] \sqrt{\dfrac{18^2+53^2+[(270+RR)/7]^2}{3}}=\sqrt{\dfrac{3133+[(270+RR)/7]^2}{3}} & \text{Al}\end{cases} \tag{1}$$

where $C$ is the criticality (%); $SR$ is the supply risk (%), which is 46 for Cu and 18 for Al from Ref. (Eheliyagoda et al., 2020); $ER$ is the environmental risk (%), which is 36 for Cu and 53 for Al from Ref. (Eheliyagoda et al., 2020); 373 and 270 from Ref. (Eheliyagoda et al., 2020); $SRR$ is supply restriction risk (%); and $RR$ is the recycling rate (%).

The formation of old scrap as product waste has the indirect potential of carbon emission reduction via an anthropogenic circularity, according to the carbon neutrality perspective. For example, in China, the three basic metals of iron, copper, and aluminum have been routinely recycled since 1949. Due to the increase in output, their total estimated emission reduction in full recycling increased exponentially from less than 1 Mt in 1949 to 1700 Mt in 2006, with iron accounting for almost 95 % of the total (Fig. 6(b)). Based on the actual recycling rate, the achieved emission reduction was expected to be between 270 Mt in 2009 and 760 Mt in 2019, with an average annual increase rate of roughly 18 %.

## 5.3 Opportunities of anthropogenic circularity

Currently, the recycling rate for the majority of metals is still less than 50 % so we are far away from a closed-loop material society (Reck and Graedel, 2012; Hool et al., 2022). As a global society, the total production of copper grew from 4.64 Mt in 1960 to 15.9 Mt in 2010. Meanwhile, old scrap played an increasing role in this production from 32 % in 1960 to 60 %–63 % in 2010 (Table 1). Nearly half of the total zinc losses in the whole life cycle occur due to the old scrap not being collected for recycling after being discarded (Rostek et al., 2022). Hence, improving this life stage can make the largest contribution towards more circularity. From a circular economy perspective, the life cycle gap in carbon emission was 54 %, 61 %, and 98 % for plastic bottles,

**Fig. 6** (a) The mathematical relations between anthropogenic circularity and metal criticality in China; (b) The potential emission reduction from typical metals production in China. Note: calculated from China's mine production and Fig. 4(b). (c) Achieved emission reduction from typical metals production in China.

**Table 1** The global copper old scrap and its potential contribution ratio in total production

| Year | Old scrap generation (Mt) (Glöser et al., 2013) | Old scrap generation (Mt) (Ali et al., 2017) | Total production (Mt)* | Ratio 1 (%) | Ratio 2 (%) |
|---|---|---|---|---|---|
| 1960 | 1.5 | 1.5 | 4.64 | 32 | 32 |
| 1965 | 1.9 | 1.8 | 5.59 | 34 | 32 |
| 1970 | 2.3 | 2.1 | 6.633 | 35 | 32 |
| 1975 | 3 | 2.5 | 7.867 | 38 | 32 |
| 1980 | 3.5 | 3 | 7.656 | 46 | 39 |
| 1985 | 4.3 | 3.5 | 8.088 | 53 | 43 |
| 1990 | 5.2 | 4.5 | 8.95 | 58 | 50 |
| 1995 | 6.1 | 5.5 | 10 | 61 | 55 |
| 2000 | 7.2 | 6.5 | 13.2 | 55 | 49 |
| 2005 | 8.5 | 7.8 | 15 | 57 | 52 |
| 2010 | 10 | 9.5 | 15.9 | 63 | 60 |

Note: *data source from USGS; Ratio 1 = Old scrap generation from the Ref. (Glöser et al., 2013) / Production × 100 %; Ratio 2 = Old scrap generation from the Ref. (Ali et al., 2017) / Production × 100 %.

rechargeable batteries, and T-shirts, respectively (Dieterle and Viere, 2022). Accordingly, there is a copious opportunity for further circularity improvements to achieve anthropogenic circularity and sustainability (Tan et al., 2021).

Circular economy and zero waste have been used to validate anthropogenic circularity in the above-mentioned practice (Chen and Ogunseitan, 2021). In recent years, the European Union and China, for example, have developed a full circular economy package or program. EoL vehicles and e-waste recycling are two examples of growing industry. The automobile sector recycles at a significantly higher rate than the e-waste industry. China launched a larger-scale trial program for a zero-waste city in 2019, with the goal of promoting all waste collection and recycling methods (Awasthi et al., 2021). After two years of success, the technology, policy, standard, and method for less generation and more collecting have been scaled up. Since 2022 China has expanded its zero-waste city program to more than 100 cities. The largest scale anthropogenic circularity in the world is embarking.

## 6 Conclusions and perspectives

Growing circularity not only minimizes environmental deterioration but also reduces energy consumption because recycling requires less energy than generating new materials. Anthropogenic circularity can help carbon neutrality by reducing metal criticality through reuse, remanufacturing, recycling, and recovery. Criticality measurement emphasizes improved recyclability and circularity. One effective solution against metal criticality was found to be anthropogenic circularity through recycling. During the entire actual circularity, China's maximum rising potential for copper and aluminum criticality was 4 % and 3 %, respectively, during the entire actual circularity. However, in 2006, China only managed to cut emissions by 15 % of their potential. Further circularity advancements have enormous promise for achieving a win-win scenario of metal criticality and carbon neutrality.

Some anthropogenic circularity initiatives must be completed to maximize the win-win vision: an advanced virgin mining procedure should be created to decrease tailings and slag in the upstream life cycle. To get rid of the historical dumpling, open cycle recycling may be required. An orderly governance of geological minerals could be established in a controlled manner; for the middle stream of a life cycle, new scrap generated during the manufacturing stage must be fully collected for recycling; for old scrap, collection remains the primary barrier to anthropogenic circularity. The new regulation and incentive should be encouraged so that all waste can be handled safely.

**Acknowledgements** This work was supported by the National Natural Sciences Foundation of China (No. 92062111) and the National Key R&D Program of China (No. 2019YFC1908501).

**Electronic Supplementary Material** Supplementary material is available in the online version of this article at https://doi.org/10.1007/s11783-023-1623-2 and is accessible for authorized users.

**Open Access** This article is licensed under a Creative Commons Attribution 4.0 International License, which permits use, sharing, adaptation, distribution and reproduction in any medium or format, as long as you give appropriate credit to the original author(s) and the source, provide a link to the Creative Commons licence, and indicate if changes were made. The images or other third party material in this article are included in the article's Creative Commons licence, unless indicated otherwise in a credit line to the material. If material is not included in the article's Creative Commons licence and your intended use is not permitted by statutory regulation or exceeds the permitted use, you will need to obtain permission directly from the copyright holder. To view a copy of this licence, visit http://creativecommons.org/licenses/by/4.0/.

## Appendix A

| Terminology | Definition |
|---|---|
| Anthropogenic circularity | Anthropogenic circularity is the human activity to enclose the material circular utilization, which is mainly consisting of reuse, remanufacturing, recycling, and recovery. |
| Anthropocene | The anthropocene is the current geological age, viewed as the period during which human activity has been the dominant influence on our earth. |
| Anthroposphere | The anthroposphere (also referred as technosphere) is that part of the environment that is made or modified by human for use in human activity and human habitats. |
| Carbon neutrality | Carbon neutrality is a state of net-zero carbon dioxide emissions. |
| Circular economy | A circular economy is an economic model designed to minimize resource input, as well as waste and emission production. |
| Criticality | Criticality is the quality, state, or degree of being of the highest importance. |
| Emergy analysis | Emergy analysis is a type of embodied energy analysis that can provide common units for comparison of environmental and economic goods by summing the energy of one type required directly or indirectly for production of goods. |
| Home scrap | Home scrap is the originated scrap that is utilized within the plant. |
| New scrap | New scrap is the solid waste generated from manufacturing process. |
| Old scrap | Old scrap is the solid waste generated from product consumption. |
| Recyclability | Recyclability is the theoretical probability of an item's being recycled. |
| Urban mining | Urban mining is the process of reclaiming raw materials from waste products sent to landfill. |

## Author Biography

Dr. Xianlai Zeng is currently an associate professor in the School of Environment at Tsinghua University, China. He obtained his Bachelor (2002) and Master (2005) degrees from Northwest A&F University, and Ph. D (2014) from Tsinghua University. He worked as a technical advisor for the United Nations Development Programme (2015), visiting staff at Coventry University (2012), visiting professor at Macquarie University (2017), and Fulbright fellow at Yale University (2018–2019). His areas of specialization and interest include waste recycling and circular economy. He proposed the emerging concept and framework of anthropogenic circularity. He has published around 100 articles and books, which have been cited for more than 8000 times with an H-index of 38 (Google Scholar). As the PI, he has chaired over 20 international and national competitive projects related to waste management, metal sustainability, and circular economy. In addition, Dr. Zeng has received 10 granted patents and led one group standard of resource and environmental properties of industrial solid waste. He has chaired or organized a dozen leading international academic meetings or workshops on material flow and waste management.

## Supporting Information

Appendix
