# Supplementary material for: Win-Win: Anthropogenic circularity for metal criticality and carbon neutrality
Source: Front Environ Sci Eng. 2022 Sep 5;17(2):23. doi: 10.1007/s11783-023-1623-2 (PMC9467426; doi:10.1007/s11783-023-1623-2)
Supplement: Supplementary file 1 — Appendix [file 11783_2023_1623_MOESM1_ESM.pdf]

# Supplementary files

**Table S1** Collected amount of research articles related to metal criticality until March 2022

| Element | Total amount | Reference |
| --- | --- | --- |
| Ag | 3 | (Nassar et al., 2012; Graedel et al., 2015; Yan et al., 2021) |
| Al | 4 | (Nassar et al., 2012; Ciacci et al., 2016; Eheliyagoda et al., 2020; Yan et al., 2021) |
| As | 3 | (Nassar et al., 2012; Graedel et al., 2015; Yan et al., 2021) |
| Au | 2 | (Nassar et al., 2012; Graedel et al., 2015) |
| B | 2 | (Graedel et al., 2015; Yan et al., 2021) |
| Ba | 2 | (Graedel et al., 2015; Panousi et al., 2016) |
| Be | 2 | (Graedel et al., 2015; Yan et al., 2021) |
| Bi | 4 | (Graedel et al., 2015; Glöser-Chahoud et al., 2016; Panousi et al., 2016; Yan et al., 2021) |
| Cd | 2 | (Graedel et al., 2015; Harper et al., 2015b) |
| Ce | 2 | (Graedel et al., 2015; Nassar et al., 2015) |
| Co | 2 | (Graedel et al., 2015; Yan et al., 2021) |
| Cr | 3 | (Nuss et al., 2014; Graedel et al., 2015; Yan et al., 2021) |
| Cs | 1 | (Yan et al., 2021) |
| Cu | 9 | (National-Research-Council, 2008; European-Commission, 2010; Erdmann and Graedel, 2011; Nassar et al., 2012; Graedel et al., 2015; Ciacci et al., 2016; Eheliyagoda et al., 2020; Helbig et al., 2021; Yan et al., 2021) |
| Dy | 3 | (Rademaker et al., 2013; Graedel et al., 2015; Nassar et al., 2015) |
| Er | 2 | (Graedel et al., 2015; Nassar et al., 2015) |
| Eu | 2 | (Graedel et al., 2015; Nassar et al., 2015) |
| Fe | 3 | (Nuss et al., 2014; Graedel et al., 2015; Ciacci et al., 2016) |
| Ga | 5 | (National-Research-Council, 2008; European-Commission, 2010; Erdmann and Graedel, 2011; Graedel et al., 2015; Yan et al., 2021) |
| Gd | 2 | (Graedel et al., 2015; Nassar et al., 2015) |
| Ge | 4 | (Graedel et al., 2015; Harper et al., 2015b; Glöser-Chahoud et al., 2016; Yan et al., 2021) |
| Hf | 2 | (Graedel et al., 2015; Harper et al., 2015a) |
| Hg | 3 | (Graedel et al., 2015; Panousi et al., 2016; Yan et al., 2021) |
| Ho | 2 | (Graedel et al., 2015; Nassar et al., 2015) |
| In | 8 | (National-Research-Council, 2008; European-Commission, 2010; Erdmann and Graedel, 2011; Graedel et al., 2015; Harper et al., 2015b; Ciacci et al., 2016; Helbig et al., 2021; Yan et al., 2021) |
| La | 2 | (Graedel et al., 2015; Nassar et al., 2015) |
| Ir | 1 | (Graedel et al., 2015) |
| Li | 5 | (National-Research-Council, 2008; European-Commission, 2010; Erdmann and Graedel, 2011; Graedel et al., 2015; Yan et al., 2021) |
| Lu | 2 | (Graedel et al., 2015; Nassar et al., 2015) |
| Mg | 3 | (Graedel et al., 2015; Glöser-Chahoud et al., 2016; Yan et al., 2021) |

| | | |
|---|---|---|
| Mn | 6 | (National-Research-Council, 2008; European-Commission, 2010; Erdmann and Graedel, 2011; Nuss et al., 2014; Graedel et al., 2015; Yan et al., 2021) |
| Mo | 2 | (Graedel et al., 2015; Yan et al., 2021) |
| Nb | 6 | (National-Research-Council, 2008; European-Commission, 2010; Erdmann and Graedel, 2011; Nuss et al., 2014; Graedel et al., 2015; Yan et al., 2021) |
| Nd | 3 | (Rademaker et al., 2013; Graedel et al., 2015; Nassar et al., 2015) |
| Ni | 3 | (Graedel et al., 2015; Ciacci et al., 2016; Yan et al., 2021) |
| Os | 1 | (Graedel et al., 2015) |
| Pb | 2 | (Graedel et al., 2015; Harper et al., 2015b) |
| Pd | 5 | (National-Research-Council, 2008; European-Commission, 2010; Erdmann and Graedel, 2011; Graedel et al., 2015; Glöser-Chahoud et al., 2016) |
| Pr | 2 | (Graedel et al., 2015; Nassar et al., 2015) |
| Pt | 6 | (National-Research-Council, 2008; European-Commission, 2010; Erdmann and Graedel, 2011; Graedel et al., 2015; Glöser-Chahoud et al., 2016; Yan et al., 2021) |
| Rb | 1 | (Yan et al., 2021) |
| Re | 2 | (Graedel et al., 2015; Yan et al., 2021) |
| Rh | 4 | (National-Research-Council, 2008; European-Commission, 2010; Erdmann and Graedel, 2011; Graedel et al., 2015) |
| Ru | 1 | (Graedel et al., 2015) |
| Sb | 3 | (Graedel et al., 2015; Panousi et al., 2016; Yan et al., 2021) |
| Sc | 2 | (Graedel et al., 2015; Panousi et al., 2016) |
| Se | 3 | (Nassar et al., 2012; Graedel et al., 2015; Yan et al., 2021) |
| Si | 1 | (Yan et al., 2021) |
| Sm | 2 | (Graedel et al., 2015; Nassar et al., 2015) |
| Sn | 3 | (Graedel et al., 2015; Harper et al., 2015b; Yan et al., 2021) |
| Sr | 3 | (Graedel et al., 2015; Panousi et al., 2016; Yan et al., 2021) |
| Ta | 2 | (Graedel et al., 2015; Yan et al., 2021) |
| Tb | 2 | (Graedel et al., 2015; Nassar et al., 2015) |
| Te | 3 | (Nassar et al., 2012; Graedel et al., 2015; Yan et al., 2021) |
| Th | 2 | (Graedel et al., 2015; Harper et al., 2015a) |
| Ti | 5 | (National-Research-Council, 2008; European-Commission, 2010; Erdmann and Graedel, 2011; Graedel et al., 2015; Glöser-Chahoud et al., 2016) |
| Tl | 2 | (Graedel et al., 2015; Panousi et al., 2016) |
| Tm | 2 | (Graedel et al., 2015; Nassar et al., 2015) |
| U | 2 | (Graedel et al., 2015; Harper et al., 2015a) |
| V | 3 | (Nuss et al., 2014; Graedel et al., 2015; Yan et al., 2021) |
| W | 3 | (Graedel et al., 2015; Glöser-Chahoud et al., 2016; Yan et al., 2021) |
| Y | 2 | (Graedel et al., 2015; Nassar et al., 2015) |
| Yb | 2 | (Graedel et al., 2015; Nassar et al., 2015) |
| Zn | 4 | (Graedel et al., 2015; Harper et al., 2015b; Ciacci et al., 2016; Yan et al., 2021) |
| Zr | 2 | (Harper et al., 2015a; Yan et al., 2021) |

**References**

Ciacci L, Nuss P, Reck B, Werner T, Graedel T (2016). Metal Criticality Determination for Australia, the US, and the Planet—Comparing 2008 and 2012 Results. Resources, 5(4): 29

Eheliyagoda D, Zeng X, Li J (2020). A method to assess national metal criticality: the environment as a foremost measurement. Humanities and Social Sciences Communications, 7: 43

Erdmann L, Graedel T E (2011). Criticality of non-fuel minerals: a review of major approaches and analyses. Environ Sci Technol, 45(18): 7620-7630

European-Commission (2010). Critical raw materials for the EU. Report of the Ad-hoc Working Group on defining critical raw materials. Brussels, Belgium

Glöser-Chahoud S, Tercero Espinoza L, Walz R, Faulstich M (2016). Taking the Step towards a More Dynamic View on Raw Material Criticality: An Indicator Based Analysis for Germany and Japan. Resources, 5(4): 45

Graedel T E, Harper E M, Nassar N T, Nuss P, Reck B K (2015). Criticality of metals and metalloids. Proceedings of the National Academy of Sciences of the United States of America, 112(14): 4257-4262

Harper E M, Diao Z, Panousi S, Nuss P, Eckelman M J, Graedel T E (2015a). The criticality of four nuclear energy metals. Resources, Conservation and Recycling, 95(0): 193-201

Harper E M, Kavlak G, Burmeister L, Eckelman M J, Erbis S, Sebastian Espinoza V, Nuss P, Graedel T E (2015b). Criticality of the Geological Zinc, Tin, and Lead Family. Journal of Industrial Ecology, 19(4): 628-644

Helbig C, Schrijvers D, Hool A (2021). Selecting and prioritizing material resources by criticality assessments. One Earth, 4(3): 339-345

Nassar N T, Barr R, Browning M, Diao Z, Friedlander E, Harper E M, Henly C, Kavlak G, Kwatra S, Jun C, Warren S, Yang M Y, Graedel T E (2012). Criticality of the geological copper family. Environ Sci Technol, 46(2): 1071-1078

Nassar N T, Du X Y, Graedel T E (2015). Criticality of the Rare Earth Elements. Journal of Industrial Ecology, 19(6): 1044-1054

National-Research-Council (2008). Minerals, critical minerals, and the US economy. Washington, D.C.: National Academies Press

Nuss P, Harper E M, Nassar N T, Reck B K, Graedel T E (2014). Criticality of Iron and Its Principal Alloying Elements. Environmental Science & Technology, 48(7): 4171-4177

Panousi S, Harper E M, Nuss P, Eckelman M J, Hakimian A, Graedel T E (2016). Criticality of Seven Specialty Metals. Journal of Industrial Ecology, 20(4): 837-853

Rademaker J H, Kleijn R, Yang Y (2013). Recycling as a Strategy against Rare Earth Element Criticality: A Systemic Evaluation of the Potential Yield of NdFeB Magnet Recycling. Environmental Science & Technology, 47(18): 10129-10136

Yan W, Wang Z, Cao H, Zhang Y, Sun Z (2021). Criticality assessment of metal resources in China. iScience, 24(6): 102524
